# Uric acid and future complications in young individuals with type 1 diabetes: results from the Diabetes Incidence Study in Sweden (DISS) and the National Diabetes Registry of Sweden (NDR)

**DOI:** 10.1007/s00125-025-06561-w

**Published:** 2025-10-14

**Authors:** Katarina Fagher, Katarina Eeg-Olofsson, Hans Arnqvist, Jan Bolinder, Jan W. Eriksson, Soffia Gudbjörnsdottir, Lennarth Nyström, Mona Landin-Olsson

**Affiliations:** 1 Department of Clinical Sciences, Lund University, Lund, Sweden; 2 Department of Endocrinology, Skåne University Hospital, Lund, Sweden; 3 The Swedish National Diabetes Registry, Centre of Registries, Gothenburg, Sweden; 4 Department of Molecular and Clinical Medicine, Institute of Medicine, University of Gothenburg, Gothenburg, Sweden; 5 DISS Study Group, Division of Endocrinology, Department of Biomedical and Clinical Sciences, Linköping University, Linköping, Sweden; 6 DISS Study Group, Department of Medicine, Karolinska University Hospital Huddinge, Karolinska Institute, Stockholm, Sweden; 7 DISS Study Group, Department of Medical Sciences, Uppsala University, Uppsala, Sweden; 8 DISS Study Group, Department of Epidemiology and Global Health, Umeå University, Umeå, Sweden; 9 DISS Study Group, Skåne University Hospital, Lund, Sweden

**Keywords:** Biomarkers, Diabetes complications, Type 1 diabetes, Uric acid

## Abstract

**Aims/hypothesis:**

The aim of this work was to investigate whether baseline uric acid (UA) is associated with future complications among young individuals with newly diagnosed type 1 diabetes.

**Methods:**

UA levels were analysed in individuals, aged 15–34 years with newly diagnosed type 1 diabetes, from the nationwide Diabetes Incidence Study in Sweden (DISS) cohort to assess the relationship with macro- and microvascular complications later in life. Information on complications was obtained by record linkage to the National Diabetes Registry of Sweden and the National Patient Registry of Sweden. Individuals who developed complications during follow-up (*n *= 94) were matched for year and age at diagnosis (±2 years), sex and HbA_1c_ with control individuals (*n *= 94) without complications.

**Results:**

Plasma UA levels at the time of diabetes diagnosis were significantly higher in individuals who later developed diabetes-related complications compared with those who did not, after a median follow-up of 19.0 years (IQR 16.3–21.0): 209.2 ± 68.9 vs 171.7 ± 50.2 µmol/l (*p<*0.001). The odds of developing complications were 1% higher for every 1 μmol/l rise in baseline UA, and individuals within the highest quartile of UA were more than three times more likely to develop diabetes-related complications later in life after adjusting for age, HbA_1c_, smoking and eGFR.

**Conclusions/interpretation:**

This study indicates that higher baseline UA levels at the time of type 1 diabetes diagnosis may be linked to both macrovascular and microvascular complications later in life.

**Graphical Abstract:**

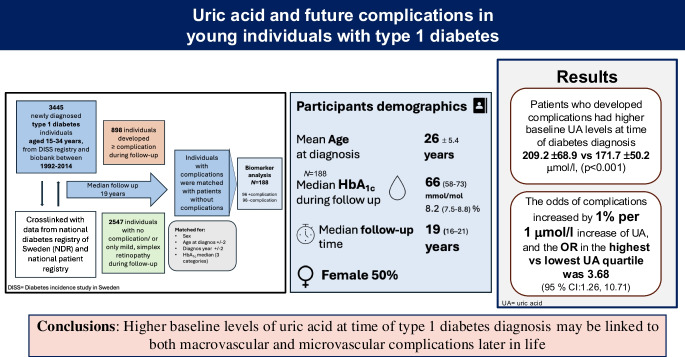


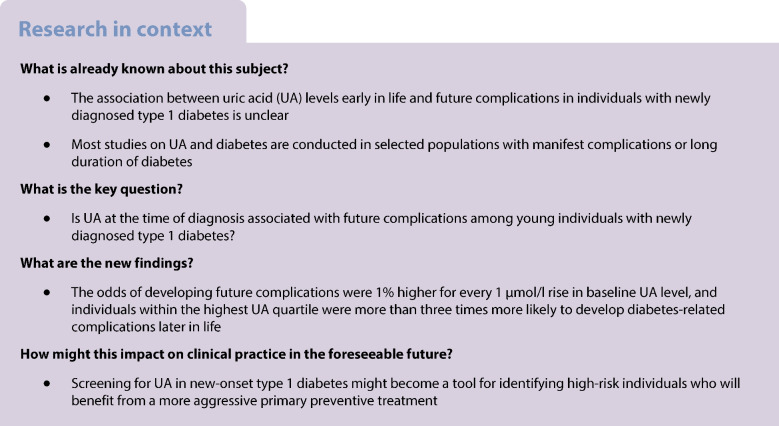


## Introduction

Currently, 6% of the Swedish population have been diagnosed with diabetes and the disease is growing into epidemic proportions worldwide [1]. Both macro- and microvascular diseases are highly over-represented among individuals with diabetes and, despite optimal metabolic regulation, the risk of, for example, CVD is doubled in diabetes compared with the background population [2]. Although the majority of CVD occurs in individuals with type 2 diabetes, considering that people with type 1 diabetes are younger at onset, they consequently risk losing more life-years from CVD [3, 4]. Traditional cardiovascular risk factors such as elevated HbA_1c_, hypertension, smoking, younger age at diagnosis and hyperlipidaemia can partly, but not alone, explain the increased risk of developing complications and the role of other factors needs further evaluation. Exploring early potential risk factors and biomarkers at the time of diabetes diagnosis might help us to improve risk stratification and might avoid premature morbidity and mortality.

Uric acid (UA) is the degradation product of purines. Increased levels of UA in plasma or in serum is widely recognised as a risk factor for both macro- and microvascular complications as well as mortality [5–7]. However, evidence on the association between UA early in life and future complications in individuals with newly diagnosed type 1 diabetes is lacking, as many studies are cross-sectional or conducted in selected populations with manifest complications or long diabetes duration. To fill this knowledge gap, we analysed UA in people aged 15–34 years with new-onset type 1 diabetes from the nationwide Diabetes Incidence Study in Sweden (DISS) cohort, to assess a plausible relationship with macro- and microvascular complications later in life.

## Methods

### Study population and recruitment of participants

**Fig. 1:**
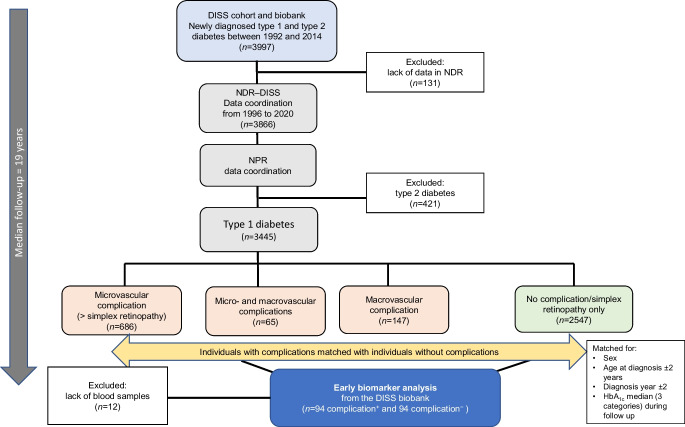
Flow chart for recruitment of individuals in the study

Since 1 January 1983 almost all incident cases of diabetes in the age group 15–34 years have been reported to the DISS on a specific form and a blood sample was taken and sent to the DISS Biobank at Lund University [8–10]. The biobank currently contains over 12,000 blood samples from individuals newly diagnosed with diabetes in Sweden between 1983 and 2014. In this study, cases of diabetes reported in the DISS registry between 1992 and 2014 (*n*=3997) (Fig. 1) were linked to the National Diabetes Register of Sweden (NDR) between 1996 and 1 January 2020, when data was collected. After exclusion of individuals with type 2 diabetes (*n*=421), 3445 individuals remained for follow-up with respect to complications. The NDR started in 1996 as a quality register of diabetes care in Sweden. Year of onset, type of diabetes, treatment, metabolic status measured as HbA_1c_, BP and diabetes-related complications are registered every year. At present, the ascertainment of registration is close to 100%.

From the DISS register, data on diabetes type, age and year of diagnosis and presence of islet antibodies were collected. From the NDR register, HbA_1c_, smoking habits, hypertension (defined as BP ≥140/90 mmHg and/or use of BP-lowering drugs), and data on complications (micro- or macroalbuminuria, nephropathy, cardiac disease, stroke, peripheral vascular disease [PVD], neuropathy and retinopathy) were collected. A median HbA_1c_ during follow-up was calculated for each individual, and individuals were categorised into three HbA_1c_ groups: ≤58 mmol/mol (≤7.5%); 59–69 mmol/mol (7.5–8.5%); and ≥70 mmol/mol (≥8.6%). Diabetes complications were also identified from the Swedish National Patient Register (NPR) of all in-hospital discharge records as well outpatient medical records using codes from the ICD 9th Revision and 10th Revision. Cause of death was obtained by record linkage to the National Cause of Death Register (NCDR) 1992–2019. A nested case–control study design was used, where each individual with type 1 diabetes who had developed at least one diabetic complication (macro- and/or microvascular) during follow-up was matched by age at diagnosis (±2 years), sex, year of diagnosis (±2 years) and HbA_1c_ (group) with two control individuals without any complications (one ordinary control individual and one reserve, where the latter was chosen only if blood samples were unavailable).

This study included both men and women with type 1 diabetes, with sex ascertained from medical records. Cases and controls were matched 1:1 on sex, given the evidence of sex-based differences in progression of complications. Gender identity was not recorded and therefore not considered in the analyses.

A power analysis estimated a sample size of 100 individuals in each group to detect a 10% difference in biomarkers with a power of 80% (β) and a two-tailed significance level of 0.05 (α). We selected 100 individuals with the highest burden of complications (i.e. individuals with complications from more than one organ system), and 100 of their matched control counterparts, for biomarker analysis. First, eligible individuals with both micro- and macrovascular complications were selected by computer together with their matched control counterparts. After that, individuals with one or more complications of either macro- or microvascular nature were selected within all three HbA_1c_ groups. Individuals were selected regardless of whether the individual died during the follow-up period or not, and mortality data was added first after selection was complete. A flow chart for recruitment of individuals in the study is given in Fig. 1.

Ethical approval was obtained by the Human Ethics Committee in Sweden (Dnr 2019–05886).

### Diabetes classification

The clinical classification of type 1 diabetes in this study was based on the presence of at least one islet antibody at diagnosis. People positive for antibody in DISS but classified as type 2 or unclassified diabetes in the NDR were classified as type 1 diabetes in our analysis.

### Outcomes

Nephropathy was defined as presence of microalbuminuria or macroalbuminuria (i.e. urinary albumin/creatinine ratio [UACR] of 3–30 mg/mmol and >30 mg/mmol, respectively), in two of three collected urine specimens) and/or a decline in renal function with eGFR <60 ml/min per 1.73 m^2^, and/or clinical ICD diagnosis of chronic kidney disease (CKD).

Since mild retinopathy (simplex retinopathy) is commonly present and sometimes resolves, we chose to not include individuals with only mild retinopathy when selecting those with manifest diabetes complications for biomarker analysis.

CVD was defined as at least one diagnosis of angina pectoris, acute coronary syndrome or myocardial infarction. Cerebrovascular disease was defined as at least one diagnosis of stroke (ischaemic or haemorrhagic) or transitory ischaemic attack (TIA).

PVD was defined as at least one diagnoses of intermittent claudication, peripheral atherosclerotic disease or peripheral gangrene.

Neuropathy was considered present among those with at least one diagnoses of peripheral neuropathy, autonomic neuropathy or focal (mono-) neuropathy.

### Analysis of early biomarkers

Blood samples in the DISS biobank were collected in tubes containing EDTA at the time of diabetes diagnosis and were stored at −80℃ in a freezer.

Plasma concentrations of UA, LDL-cholesterol, HDL-cholesterol, creatinine, cystatin and C-reactive protein (CRP) were analysed at the local accredited laboratory at Skåne University Hospital in Lund, Sweden. Baseline eGFR was calculated using the CKD-EPI equation [11].

### Statistical analysis

We used the software program MatchIt (version 4.3.4) for matching participants for biomarker analysis [12]. All analyses were performed using either Microsoft Excel (Microsoft, Redmond, WA, USA) or SPSS version 26 (IBM, NY, USA). Shapiro–Wilk’s test, and visual inspection of histograms and Q-Q plots were performed to test data for normality [13]. Normally distributed data were expressed as mean ± SD and non-normally distributed as median (IQR or range [minimum–maximum]).

**Fig. 2:**
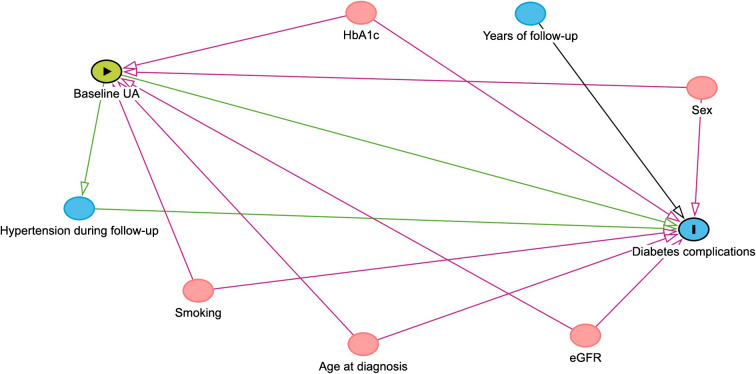
DAG illustrating the presumed causal relationships between UA and diabetes complications. UA is the exposure variable and diabetes complications represent the outcome. Variables highlighted in pink are plausible confounders, as they influence both the exposure and the outcome. The remaining variables, shown in blue, affect only the outcome and are therefore not classified as confounders

Differences between matched individuals were tested with Student’s paired test for normally distributed data, otherwise Wilcoxon signed rank test was used. McNemar’s test was used when comparing categorical variables between matched individuals. A two-tailed *p*<0.05 was considered statistically significant. UA was tested both as a continuous variable and as a categorised variable (quartiles) in a logistic regression analysis, to estimate the likelihood expressed as OR with 95% CI for developing future complications. To identify potential confounders for inclusion in the multivariable analysis, we constructed a directed acyclic graph (DAG) (Fig. 2) [14]. All potential confounders identified in the DAG were included in the multivariable regression analysis, except for sex, which was matched 1:1 between individuals.

## Results

In total, 3445 individuals with newly diagnosed type 1 diabetes from the DISS registry were followed for a median of 19.0 years (IQR 16.3–21.0) (Fig. 1). Of these, 898 developed at least one diabetes-related complication. Microvascular complications were most common (*n*=686), of which retinopathy (defined as > mild retinopathy) was the most frequently reported (476 events) followed by nephropathy (395 events). In total, 339 individuals died during follow-up.

Of the 200 matched individuals, 12 (six with complications, together with their matched control counterparts) were excluded due to failed blood sample analysis. Clinical characteristics and distribution of complications for the remaining 188 individuals are summarised in Table 1. The proportion of macro- and microvascular complications within the study population were as follows: 42 individuals had both macro- and microvascular complications; 18 had macrovascular complications only; and 34 had complications of a microvascular nature. There were small but significant differences in baseline demographics between the matched individuals, regarding age at diagnosis (0.6 years older in the complication group), follow-up time and HbA_1c_, as demonstrated in Table 1. Further, individuals who developed complications had a higher prevalence of hypertension, as well as higher mean systolic BP during follow-up. At the onset of diabetes, plasma levels of UA and cystatin C were significantly higher in individuals who later developed complications compared with those without complications (Table 2). The following variables, identified as potential confounders in the DAG, were included in the multivariable regression analysis: age (categorised in intervals of 5 years); smoking; HbA_1c_; and eGFR. Both unadjusted and adjusted regression models showed that higher baseline UA levels were significantly associated with higher odds of developing future complications. This association was consistent across both microvascular and macrovascular complications, as demonstrated in Table 3. The odds of complications were 1% higher per 1 μmol/l increase in baseline UA level, and the OR in the highest compared with lowest UA quartile was 3.68 (95% CI 1.26, 10.71) for future complications of any nature. For micro- and macrovascular complications, the OR was 4.13 (95% CI: 1.34, 12.78) and 9.26 (95% CI: 2.14, 40.15), respectively.

**Table 1:** Clinical characteristics and distribution of complications among individuals with complications and their matched control counterparts

Characteristic	Complication^−^ (*n*=94)	Complication^+^ (*n*=94)	*p* value
Age at diabetes diagnosis, years	26.1±5.5	26.7±5.4	<0.004^a^
HbA_1c_ category, % (*n*)			0.500^b^
≤58 mmol/mol (≤7.5%)	27.7 (26)	26.6 (25)	
59–69 mmol/mol (7.5–8.5%)	36.2 (34)	37.2 (35)	
≥70 mmol/mol (≥8.6%)	36.2 (34)	36.2 (34)	
HbA_1c_ during follow-up, mmol/mol	66 (58–72)	66 (58–76)	<0.001^c^
HbA_1c_ during follow-up, %	8.2 (7.5–8.7)	8.2 (7.5–9.1)	
Female sex, %	50	50	1.000^b^
Follow-up, years	19 (16–20)	19 (17–21)	0.016^c^
Hypertension during follow-up, %	28.7	55.3	0.001^b^
Systolic BP during follow-up (mmHg)	122.0±9.2	126.2±9.8	0.003^a^
Smoker (ever), %	37.2	48.9	0.211^b^
Complication, %			
Neuropathy	0	44.7	NA
PVD	0	23.4	NA
CVD	0	26.6	NA
Stroke	0	13.8	NA
CKD	0	53.2	NA
Retinopathy >mild/simplex, %	0	34.0	NA
Died during follow-up, % (*n*)	1.1 (1)	8.5 (8)	0.039^b^

Continuous data are given as mean ± SD or median (IQR) and categorical data as percentages or % (*n*)

^a^Student’s paired test

^b^McNemar’s test

^c^Wilcoxon signed rank test

Complication^−^, individuals without complications during follow-up; Complication^+^, individuals who developed at least one diabetes-related complication during follow-up

**Table 2:** Biomarker analysis at time of type 1 diabetes diagnosis in people with complications and their matched controls

Biomarker	Complication^−^ (*n*=94)	Complication^+^ (*n*=94)	*p* value
p-Creatinine, μmol/l	57±13	59±19	0.340^a^
p-LDL-cholesterol, mmol/l	2.5±0.9	2.7±0.8	0.276^a^
p-CRP, mg/l	4 (3–39)	4 (3–173)	0.843^b^
p-HDL-cholesterol, mmol/l	1.03±0.25	0.96±0.25	0.058^a^
p-UA, μmol/l	171.7±50.2	209.2±68.9	<0.001^a^
p-Cystatin C, mg/l	0.81±0.18	0.91±0.23	0.003^a^
eGFR, ml/min per 1.73 m^2^	115 (108–142)	117 (99−137)	0.107^b^

Values are presented as mean ± SD, except for eGFR, which is presented as median (IQR) and p-CRP, which is presented as median (range, minimum–maximum)

^a^Student’s paired test

^b^Wilcoxon signed rank test

Complication^−^, individuals without complications during follow-up; Complication^+^, individuals who developed at least one diabetes-related complication during follow-up

**Table 3:** OR (95%) of developing diabetes-related complications (any) and microvascular or macrovascular complications

	Any complication (*n*=94 complication^+^ and 94 complication^−^)		Microvascular complication (*n*=76 complication^+^ and 76 complication^−^)		Macrovascular complication (*n*=60 complication^+^ and 60 complication^−^)	
	Unadjusted	Multivariable adjusted^a^	Unadjusted	Multivariable adjusted^a^	Unadjusted	Multivariable adjusted^a^
UA, per 1 μmol/l increase	1.01 (1.00, 1.02)**	1.01 (1.00, 1.02)**	1.01 (1.00, 1.02)**	1.01 (1.00, 1.02)**	1.01 (1.00, 1.02)**	1.01 (1.01, 1.02)**
UA quartile^b^						
Quartile 1	1.0 (ref.)	1.0 (ref.)	1 (ref.)	1 (ref.)	1 (ref.)	1 (ref.)
Quartile 2	0.90(0.36, 2.25)	1.06 (0.40, 2.82)	0.80 (0.29, 2.19)	0.95 (0.32, 2.79)	2.11 (0.64, 6.99)	2.84 (0.78, 10.39)
Quartile 3	1.52 (0.63, 3.71)	1.83 (0.71, 4.73)	1.43 (0.55, 3.73)	1.66 (0.59, 4.65)	2.29 0.67, 7.76)	3.27 (0.86, 12.40)
Quartile 4	3.09 (1.24, 7.69)*	3.68 (1.26, 10.71)*	3.57 (1.38, 9.26)**	4.13 (1.34, 12.78)*	5.49 (1.65, 18.24)**	9.26 (2.14, 40.15)**

^a^The multivariable analyses were adjusted for age (5 year categories), HbA_1c_, smoking (yes [ever]/no) and eGFR

^b^Quartile 1, <180 μmol/l; quartile 2, 150–179 μmol/l; quartile 3, 180–217 μmol/l, quartile 4, >217 μmol/l

^*^p<0.05, **p<0.01

The regression model was not tested with hypertension as a covariate during follow-up, since we identified that hypertension is more likely to act as a mediator rather than a confounder (Fig. 2), given the existing evidence suggesting that UA may influence endothelial function and contribute to the development of hypertension [15, 16]. The length of follow-up was also not considered a confounder, as it is unlikely to influence UA levels at the time of diagnosis. All individuals in our study with baseline UA >400 μmol/l developed a future diabetes-related complication.

## Discussion

Our study demonstrates that plasma UA was significantly higher in individuals aged 15–35 years newly diagnosed with type 1 diabetes who later developed complications, and that higher levels of UA were independently associated with both macro- and microvascular complications later in life. This indicates that screening for UA in new-onset type 1 diabetes might become a tool for identifying high-risk individuals who will benefit from a more aggressive primary preventive treatment.

It has been debated whether hyperuricaemia is only a marker for ongoing complications or whether it is directly pathogenic. It is well-known that UA levels increase as renal function deteriorates, due to less excretion of purine metabolites in the urine [17]. In our study we found that UA was associated with future complications independent of renal function at baseline, indicating that UA might serve as an independent risk factor when risk-stratifying people with newly diagnosed type 1 diabetes.

Several observational studies have demonstrated an almost linear association between UA levels and various outcomes, including CKD [18–20], progression to end-stage kidney disease [21], cardiovascular events and death [22]. Additionally, among individuals with diabetes, a recent large study demonstrated that high levels of UA are associated with higher risk of all-cause and CVD mortality [5]. Further, in a trial including individuals with type 1 diabetes, higher UA levels were associated with a higher risk of decline in kidney function, CVD and mortality, independently of other risk factors [7]. In that trial, however, the mean diabetes duration at baseline was more than 30 years and, to our knowledge, our trial is the first to evaluate baseline UA at the time of diabetes diagnosis as a risk factor for future complications. As many previous studies investigating the role of UA and diabetes complications are cross-sectional in their design and only few longitudinal studies exist, often including older adults with a high risk of preexisting cardiovascular conditions, it has been difficult to explain a causative relationship between high levels of UA and disease progression. Our result, however, elucidates an association between UA in a younger population with newly diagnosed type 1 diabetes and no cardiorenal conditions at baseline, and the odds of developing complications later in life. Moreover, we found that this association was independent of HbA_1c_ level, renal function at baseline, smoking, sex and age. The role of UA as a biologically active mediator of complications has been advocated in several studies. Hyperuricaemia can for example induce high BP through activation of the renin–angiotensin system, either directly by decreasing neuronal nitric oxide synthesis in the juxtaglomerular apparatus or indirectly through reduced renal perfusion due to the induction of cyclooxygenase-2 in the macula densa and arterioles [15, 16], and might contribute to endothelial dysfunction, as noted by a reduction in nitric oxide metabolites [23]. In animal studies, experimental hyperuricaemic rats developed systemic hypertension as well as increasing signs of arteriopathy, tubulointestinal fibrosis and glomerulosclerosis [15, 24]. Given these experimental studies, one might postulate that lowering of UA would be effective in treating for example high BP and preventing complications. In hyperuricaemic adolescents (aged 11–17 years) with newly diagnosed hypertension, treatment with allopurinol significantly reduced BP [25]. Other studies have demonstrated similar benefits in hyperuricaemic adults with normal renal function [26]. In our study, both higher baseline UA levels and hypertension during follow-up were more common among individuals with complications, suggesting that hypertension may act as a potential mediator of future complication.

In a study by Sonoda et al [27], a significant association was observed between elevated levels of UA and future development of CKD and single-centre trials have shown that UA-lowering treatment may slow the progression of CKD [28–30]. Despite these findings, results from clinical trials of UA-lowering treatment have been inconsistent, with several studies failing to demonstrate a clear benefit. For example, in an RCT comparing allopurinol (*n*=267) with placebo (*n*=263) in people with type 1 diabetes and mild to moderate diabetic kidney disease (mean eGFR 68.0±16.9 ml/min per 1.73 m^2^), no clinical benefits were seen regarding kidney outcomes after 3 years of treatment [31]. However, if UA promotes kidney damage during long-term exposure, one could, of course, speculate that a 3 year study with a relatively small study population is too short and underpowered to reveal differences between groups. A similar result was seen in an RCT evaluating allopurinol in people with more severe kidney disease (stage 3 and 4) [32]. In that study, 182 individuals received allopurinol and 181 received placebo and no significant different was seen between groups regarding progression of kidney disease after 2 years. The study was, however, underpowered due to insufficient enrolment and a high withdrawal rate, both of which might have affected outcome. A recent subanalysis of the DAPA-HF study, a trial primarily investigating the effects of the sodium–glucose cotransporter 2 (SGLT2)-inhibitor dapagliflozin on heart failure in individuals with and without type 2 diabetes, found that dapagliflozin significantly reduced UA by 50.0 μmol/l (0.84 mg/dl) over 12 months (*p*<0.001) compared with placebo. They also found that every 60 μmol/l (1 mg/dl) increase of UA increased the risk of hospitalisation for heart failure and cardiovascular death by 7% and 6%, respectively (*p*=0.04 and *p*=0.07, respectively) [33].

Our study demonstrates that a higher baseline level of UA in young individuals with newly diagnosed type 1 diabetes is associated with future complications later in life. This finding might help us to identify individuals who would benefit from a more intensive risk factor management to prevent complications. One strength of our study is the long follow-up time for our cohort of individuals with new-onset type 1 diabetes. Further, clinical classification of type 1 diabetes was reliable in this study as it was based on presence of islet antibody at diagnosis. Another strength is the matched case–control study design, reducing the risk of bias due to different covariates. Matching on sex helped minimise confounding by biological differences. We did not, however, assess whether associations differed between men and women or explore gender-related factors, which limits generalisability across all sexes and genders. Despite matching for sex, HbA_1c_ (categorised into three groups), age at diagnosis (±2 years) and year of diagnosis (±2 years), only sex achieved a perfect match in our cohort, when analysing differences between matched individuals. This limitation is likely attributed to the small sample size, as well as the relatively young age of our study population, which results in infrequency of major macrovascular and microvascular complications. Nevertheless, baseline UA remained independently associated with future complications even after adjusting for differences in baseline characteristics, thereby reducing, though not entirely limiting, the potential impact of confounding bias. We also cannot rule out the influence of other potential confounders that were not accounted for in our analyses. Another limitation of the study is its observational design, which prevents us from establishing a causal relationship between baseline UA levels and future diabetes-related complications. Additionally, our data do not allow us to assess longitudinal changes in UA levels during follow-up or to evaluate whether medications aimed at lowering UA may have influenced complication rates. Further, we are unable to draw conclusions regarding the role of specific antihypertensive medications, such as diuretics and beta-blockers, and their potential influence on UA levels during follow-up. Moreover, complication data were obtained exclusively from national registries, raising the possibility that misclassification or misdiagnosis may have affected the reported event rates.

In conclusion, despite the limitations of our study design, our novel finding suggests that higher baseline level of UA at the time of type 1 diabetes diagnosis may be linked to both macrovascular and microvascular complications later in life. Clinical trials are needed to determine the potential effects of lowering UA on future complications risks.

## Data Availability

Data presented in the analysis are available on request from the corresponding author.

## Abbreviations

CKD: Chronic kidney disease
DAG: Directed acyclic graph
DISS: Diabetes Incidence Study in Sweden
NDR: National Diabetes Register of Sweden
PVD: Peripheral vascular disease
UA: Uric acid

## Authors’ relationships and activities

KF has received honoraria for consulting and/or lecture fees from Sanofi, Astra Zeneca, Novo Nordisk, Bayer and Amarin, all outside the submitted work. KEO has received fees for lecturing and/or honoraria for consulting from Sanofi, Novo Nordisk, Eli Lilly and Abbot Diabetes Care, all outside the submitted work. JB has received honoraria for consulting and/or lecture fees from Abbott Diabetes Care, Nanexa, Novo Nordisk and Sanofi, all outside the submitted work. The authors declare that there are no other relationships or activities that might bias, or be perceived to bias, their work.
